# A Case of Marfan Syndrome With Congenital Hip Dysplasia and Spine Abnormality

**DOI:** 10.7759/cureus.57569

**Published:** 2024-04-03

**Authors:** Siddhartha Yadao, Kartik Bansal, Shrutika M Mungal, Avni Gakkhar, Ashok M Mehendale

**Affiliations:** 1 Preventive Medicine, Jawaharlal Nehru Medical College, Datta Meghe Institute of Higher Education & Research, Wardha, IND; 2 Physiotherapy, Jawaharlal Nehru Medical College, Datta Meghe Institute of Higher Education & Research, Wardha, IND

**Keywords:** family, musculoskeletal, syndrome, abnormality, disorder

## Abstract

Marfan syndrome, a hereditary disorder of connective tissue marked by FBN1 gene mutations, presents a clinical tapestry requiring a multidisciplinary approach for optimal management. This case report details the presentation of an 11-year-old male exhibiting musculoskeletal deformities, notably an abnormally curved spine and congenital hip dysplasia, indicative of Marfan syndrome. The absence of cardiovascular abnormalities and family history challenges the diagnostic process. Clinical evaluation revealed classical signs, including positive wrist and thumb signs, pectus carinatum, a loose skin fold, and scapular winging. Laboratory investigations, including imaging studies, confirmed the diagnosis. The patient’s management involves a multifaceted strategy, addressing cardiovascular risks through beta-blockers and potential surgical interventions, orthopedic measures for musculoskeletal complications, and ophthalmologic interventions for ocular manifestations. Genetic counseling facilitates informed decision-making, and psychosocial support ensures holistic care. This case underscores the necessity of recognizing atypical presentations and employing a holistic, collaborative approach for early diagnosis and effective management of Marfan syndrome, thereby emphasizing the importance of ongoing research and heightened clinical awareness in enhancing outcomes for individuals living with this intricate genetic disorder.

## Introduction

Marfan syndrome is a multisystemic, hereditary disorder characterized by abnormalities in connective tissue, predominantly affecting the cardiovascular, skeletal, and ocular systems. First described by the French physician Antoine Marfan in 1896, this syndrome has since been recognized as one of the most common inherited connective tissue disorders, with an estimated incidence of 1 in 5,000 individuals worldwide. While the exact prevalence may vary among different populations and regions, Marfan syndrome remains a significant clinical entity with profound implications for affected individuals and their families [1]. The underlying pathophysiology of Marfan syndrome stems from mutations in the fibrillin-1 gene (FBN1), located on chromosome 15q21.1, which encodes for fibrillin-1, a significant component of microfibrils found in connective tissue [2]. These mutations lead to structural abnormalities in the extracellular matrix, resulting in systemic manifestations characteristic of the syndrome. Furthermore, approximately 25-30% of cases are due to de novo mutations with no family history of the disorder, underscoring the importance of recognizing sporadic cases in clinical practice [3]. Clinically, Marfan syndrome presents with a broad spectrum of features, varying in severity and age of onset [4]. Cardinal manifestations typically involve the musculoskeletal, cardiovascular, and ocular systems, although other organ systems may be affected to varying degrees. Skeletal abnormalities, such as tall stature, arachnodactyly (long, slender fingers), joint hypermobility, and spinal deformities, are often among the earliest signs observed in affected individuals. These skeletal manifestations can result in functional limitations and orthopedic complications, contributing to the morbidity associated with the syndrome [5]. Cardiovascular involvement represents a significant concern in Marfan syndrome, with a predisposition to aortic root dilation, aortic dissection, and valvular abnormalities. Aortic root dilation, in particular, poses a substantial risk of life-threatening complications, emphasizing the importance of regular cardiac surveillance and timely interventions to mitigate the risk of aortic dissection [1,2]. Additionally, ocular manifestations, including lens dislocation, myopia, and retinal detachment, further contribute to the clinical complexity of Marfan syndrome, necessitating comprehensive ophthalmologic evaluation and management [6]. Despite the advancements in our understanding of the genetic basis and clinical manifestations of Marfan syndrome, challenges remain in achieving early diagnosis and implementing appropriate management strategies [3]. This particular case requires timely recognition and intervention, especially by orthopedics, spine specialists, physiotherapists, and psychiatrists, as well as genetic counseling [4-6].

## Case presentation

**Figure 1:**
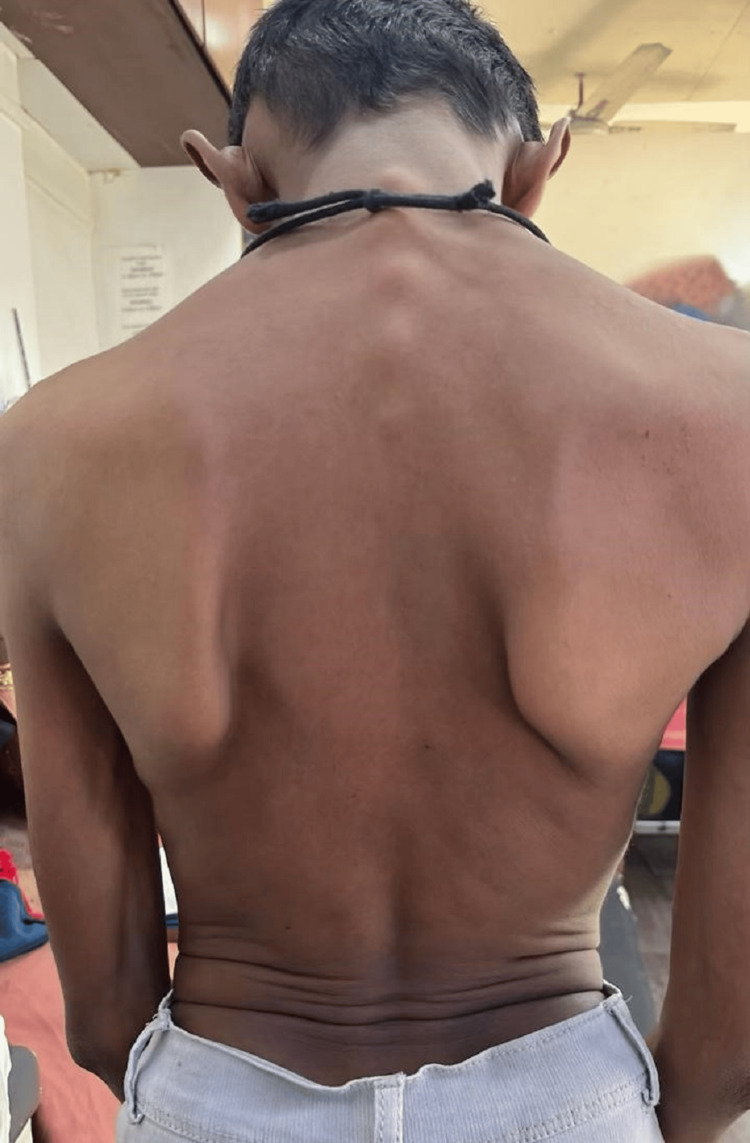
Prominent vertebrae, winging of the scapula, and a loose skin fold

**Figure 2:**
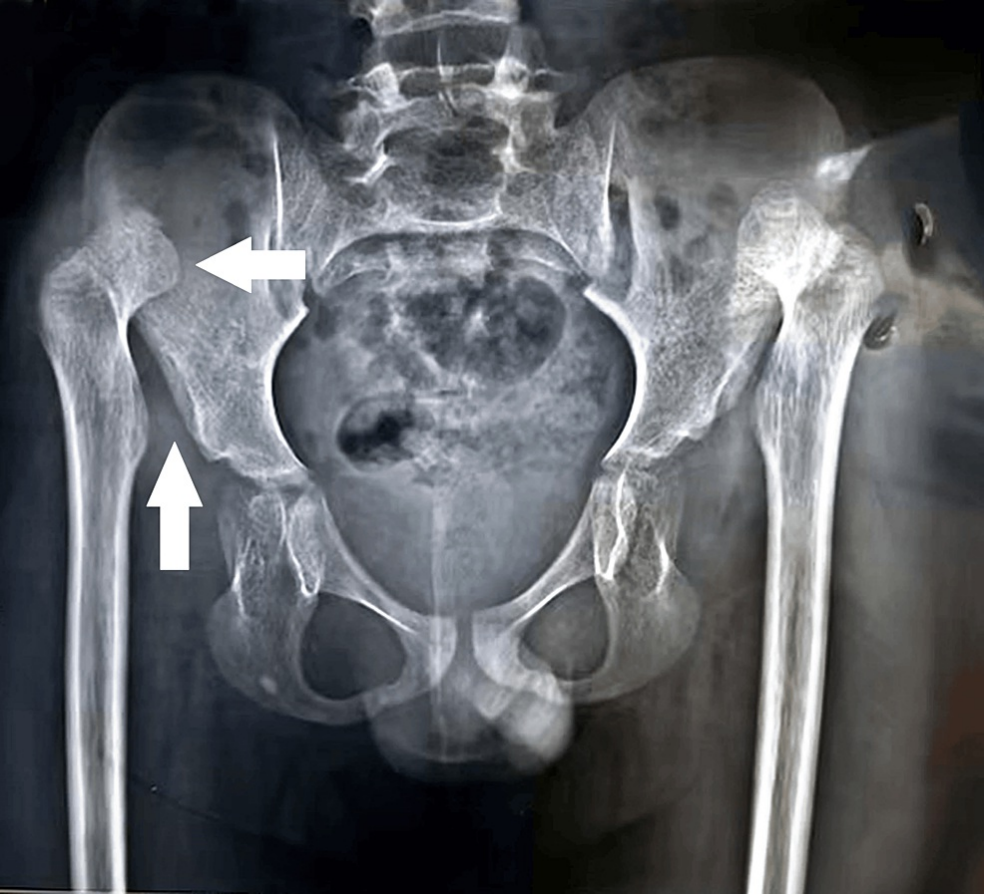
X-ray pelvis shows congenital hip dysplasia

**Figure 3:**
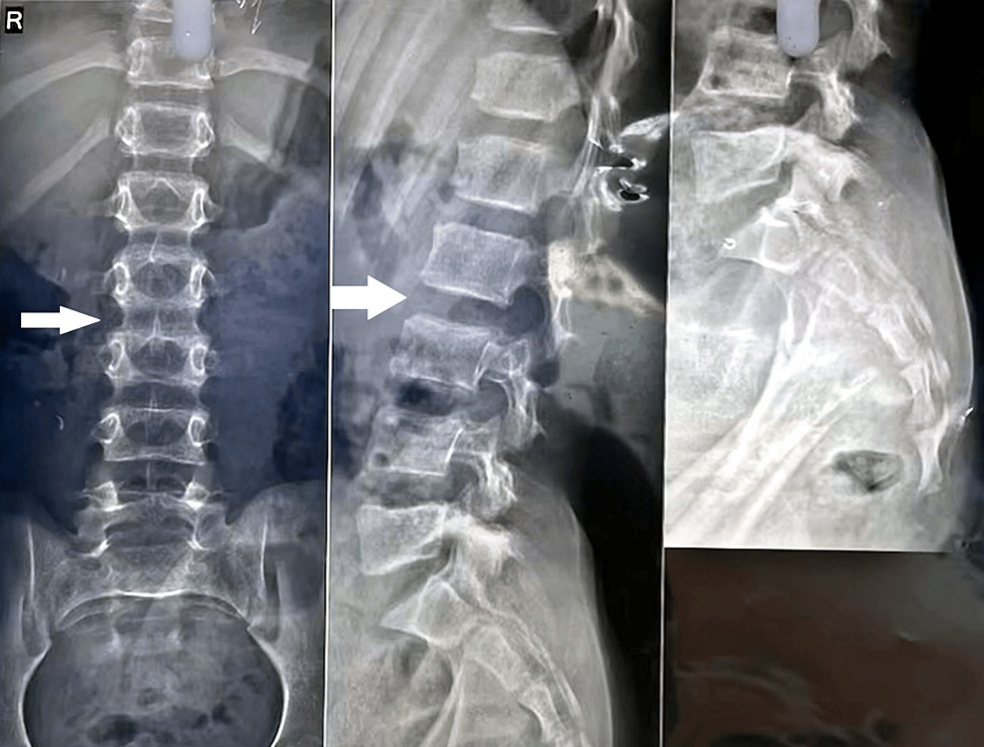
X-ray spine shows increased intervertebral disc spaces

An 11-year-old male presented with a chief complaint of difficulty walking and bearing weight since childhood, associated with an abnormally curved spine. The patient’s medical history revealed no palpitations, autonomic abnormalities, pain, or respiratory distress. Additionally, there was no familial history of similar symptoms or known genetic disorders. On physical examination, the patient exhibited a tall, thin build with musculoskeletal deformities, including positive wrist and thumb signs, pectus carinatum, a loose skin fold, and winging of the scapula (Figure 1). The general examination was otherwise normal despite these findings, and no cardiovascular abnormalities were observed. The respiratory examination demonstrated pectus carinatum. Laboratory investigations, including a complete blood count, blood urea and electrolytes, liver and kidney function tests, growth hormone, and troponin, were within normal limits. However, a chest X-ray revealed an increased cardiothoracic ratio, and an X-ray of the pelvis indicated congenital hip dysplasia (Figure 2). An X-ray of the spine demonstrated a prominent vertebral process, increased disc space, and winging of the scapula (Figure 3). Based on the clinical features and imaging findings, the patient was diagnosed with Marfan syndrome. While the absence of cardiovascular abnormalities at this stage was noted, regular cardiac monitoring was recommended due to the progressive nature of the syndrome.

## Discussion

The management of Marfan syndrome is complex and necessitates a multidisciplinary approach to address the diverse clinical manifestations affecting multiple organ systems. The primary goals of management involve preventing cardiovascular complications, optimizing musculoskeletal health, and addressing ocular abnormalities. A tailored and comprehensive care plan involving a team of specialists is essential to improve the quality of life for individuals with Marfan syndrome [7]. Cardiovascular management includes regular cardiac monitoring in individuals with Marfan syndrome due to the inherent risk of aortic root dilation and aortic dissection. Beta-blockers, such as propranolol or atenolol, are commonly prescribed to reduce the rate of aortic dilation and lower the risk of dissection. Angiotensin receptor blockers may also be considered, either alone or in combination with beta-blockers, for those who cannot tolerate beta-blockers or require additional blood pressure control. Surgical intervention, such as aortic root replacement, may be indicated in severe cases to prevent catastrophic cardiovascular events [8]. Musculoskeletal management involves managing orthopedic complications, including scoliosis, joint hypermobility, and pectus deformities, which are common in Marfan syndrome. Physical therapy and regular exercise are crucial in improving musculoskeletal function and preventing complications. Orthopedic interventions, such as bracing for scoliosis or surgical correction of pectus deformities, may be considered based on the severity of symptoms and functional impairment. Regular orthopedic follow-up is essential to monitor skeletal development and intervene as needed [9]. Ocular management involves ophthalmologic evaluation in individuals with Marfan syndrome to detect and manage ocular manifestations such as lens dislocation, myopia, and retinal detachment. Corrective lenses and surgery may be recommended to address visual impairment. Routine eye examinations are essential for ongoing monitoring and early intervention [10]. Genetic counseling is integral for individuals with Marfan syndrome and their families to understand the genetic basis of the condition, discuss inheritance patterns, and make informed family planning decisions. Psychosocial support is also crucial, as living with a chronic condition can impact mental well-being. Support groups and counseling services can provide valuable resources for individuals and their families [8-10].

## Conclusions

The management of Marfan syndrome demands a comprehensive and coordinated approach to address its diverse clinical manifestations. A multidisciplinary team involving cardiologists, orthopedic specialists, ophthalmologists, and genetic counselors is essential for effectively caring for individuals with Marfan syndrome. Cardiovascular complications can be mitigated by emphasizing regular cardiac monitoring, medical interventions, and surgical procedures when necessary. Musculoskeletal management, encompassing physical therapy, exercise, and orthopedic interventions, aims to optimize functional outcomes. Ocular assessments and interventions contribute to maintaining visual health. Furthermore, genetic counseling and psychosocial support play pivotal roles in providing information, facilitating informed decision-making, and addressing the emotional aspects of living with a chronic genetic condition. By combining medical expertise, ongoing surveillance, and individualized care plans, the management of Marfan syndrome strives to enhance the quantity and quality of life for affected individuals. Continued research and awareness efforts are crucial for refining therapeutic strategies and ensuring the best possible outcomes for those living with this complex genetic disorder.
